# Use of the sagittal Cobb* angle to guide the rod bending in the treatment of thoracolumbar fractures: a retrospective clinical study

**DOI:** 10.1186/s13018-020-02115-5

**Published:** 2020-12-01

**Authors:** Zongpo Shi, Gang Wang, Zhen Jin, Tao Wu, Haoran Wang, Jinpeng Sun, Yap San Min Nicolas, K. C. Rupesh, Kaixiang Yang, Jun Liu

**Affiliations:** Department of Orthopedics, The Second Affiliated Hospital of Nanjing Medical University, 121 Jiangjiayuan Road, Nanjing, 210000 Jiangsu China

**Keywords:** Thoracolumbar fracture, Sagittal Cobb* angle, Bending rods, Spinal sagittal parameters, Adjacent segment degeneration

## Abstract

**Background:**

Pedicle screw fixation is a well-established technique for thoracolumbar fracture. A large number of studies have shown that the bending angle of the connecting rod has a significant correlation with the postoperative spinal stability. However, no studies have confirmed an objective indicator to guide the bending angle of the connecting rod during the operation. Our study aims to define a sagittal Cobb* angle to guide the bending angle of the connecting rod during surgery.

**Methods:**

The frontal and lateral X-ray films in 150 cases of normal thoracolumbar spine were included to measure the normal spinal sagittal Cobb* angle in each segment. The patients who underwent single segment thoracolumbar fractures and pedicle screw internal fixation surgery were included. The radiological parameters included lumbar lordosis (LL), thoracic kyphosis (TK), pelvic tilt (PT), pelvic incidence (PI), sagittal vertical axis (SVA), and sacral slope (SS) were measured. The incidence of adjacent segment degeneration (ASD) 2 years after surgery was measured.

**Results:**

The average values of normal sagittal Cobb* angle in each segment were − 5.196 ± 3.318° (T12), 2.279 ± 3.324° (L1), 7.222 ± 2.798° (L2), and 12.417 ± 11.962° (L3), respectively. The LL in the three groups was 35.20 ± 9.12°, 46.26 ± 9.68°, and 54.24 ± 15.31°, respectively. Compared with the normal group, there were significant differences in group A and group C, respectively (*p* < 0.05). The results were similar in the parameters of TL, PT, and SS. The incidences of SVA > 50 mm in group A, group B, and group C were 23.33%, 12.50%, and 19.23%, respectively. The parameter of PI in three groups was 41.36 ± 12.69, 44.53 ± 15.27, and 43.38 ± 9.85°, respectively. The incidences of ASD in group A, group B, and group C 2 years after surgery were 21.67%, 13.75%, and 17.95%, respectively.

**Conclusions:**

The study confirmed that the sagittal Cobb* angle can be used as a reference angle for bending rods. When the bending angle of the connecting rod is 4 to 8° greater than the corresponding segment sagittal Cobb* angle, the patient’s spinal sagittal stability is the best 2 years after the operation.

## Introduction

Thoracolumbar fracture is most common trauma in spine surgery and is usually a high-energy trauma caused by a traffic accident or fall [1–3]. The thoracolumbar fracture has a high risk for complications including paralysis, pain, deformity, and loss of function [4]. With the wide application of pedicle screw fixation technology, it has become a reliable method in the treatment of thoracolumbar fractures [5].

Although pedicle screw fixation is a well-established technique, there are still some unsolved and neglected problems. For example, in the pedicle screw fixation technique, the bending angle of the connecting rod mainly depends on the surgeon’s experience after the pedicle screw is inserted. A large number of studies have shown that the bending angle of the connecting rod has a significant correlation with the postoperative spinal stability [6]. Moufid et al. and Glassman et al. compared the correlation between the spinal sagittal parameters and the bending angle of the connecting rod and confirmed that the inappropriate bending angle of the connecting rod is an important risk factor for adjacent segment diseases (ASD) even in short-segment fixation [7, 8]. Too large or too small rod bending angles will lead to postoperative pain, instability of the spine, adjacent segment degeneration, or other complications [9, 10].

However, no studies have confirmed an objective indicator to guide the bending angle of the connecting rod during the operation. Our study aims to define a sagittal Cobb* angle to guide the bending angle of the connecting rod during surgery. This study analyzed the influence of the difference between the rod bending angle after pedicle screw fixation of thoracolumbar fractures and the normal sagittal Cobb* angle on the postoperative spinal stability and adjacent segment degeneration, to confirm that the sagittal Cobb* angle can be used as an objective indicator to guide the bending angle of the connecting rod.

## Material and methods

The study was single-centric and retrospective. All patients who underwent single segment thoracolumbar fractures and pedicle screw internal fixation surgery in the second affiliated hospital of Nanjing Medical University during March 2015–June 2017 were included. All cases were taken the full-length spinal x-rays 2 years after surgery. Exclusion criteria were as follows: (1) significantly degenerative lordosis, kyphosis, and scoliosis; (2) the “double line shadow” of pedicle or the posterior margin of the vertebral body more than 5 mm; (3) intervertebral space stenosis; and (4) other factors that cause obvious spinal instability. The general data including age, gender, and fractured part were collected in Table 1. All patients were divided into three groups due to the different angle of bending rod (group A, the normal spinal sagittal Cobb* angle + 0 to 4°; group B, the normal spinal sagittal Cobb* angle + 4 to 8°; group C, the normal spinal sagittal Cobb* angle +8 to 12°).

**Table 1:** General data of patients

Parameter	Normal	A	B	C
Age (year, *x* ± *s*)	48.5 ± 3.6	57.5 ± 3.6	51.8 ± 2.8	55.6 ± 6.1
Sex (M/F, *n* (%))	M 62 (41.3)/F 88 (58.7)	M 22 (36.7)/F 38 (63.3)	M 32 (40.0)/F 48 (60.0)	M 33 (42.3)/F 45 (57.7)
Time of injury (day, *x* ± *s*)	―	4.7 ± 1.5	4.5 ± 2.1	5.2 ± 1.8
Segment				
T12	―	12	18	17
L1	―	17	21	21
L2	―	16	21	20
L3	―	15	20	20
Total	150	60	80	78

Time of injury indicated the mean days from injury to operation

**Fig. 1:**
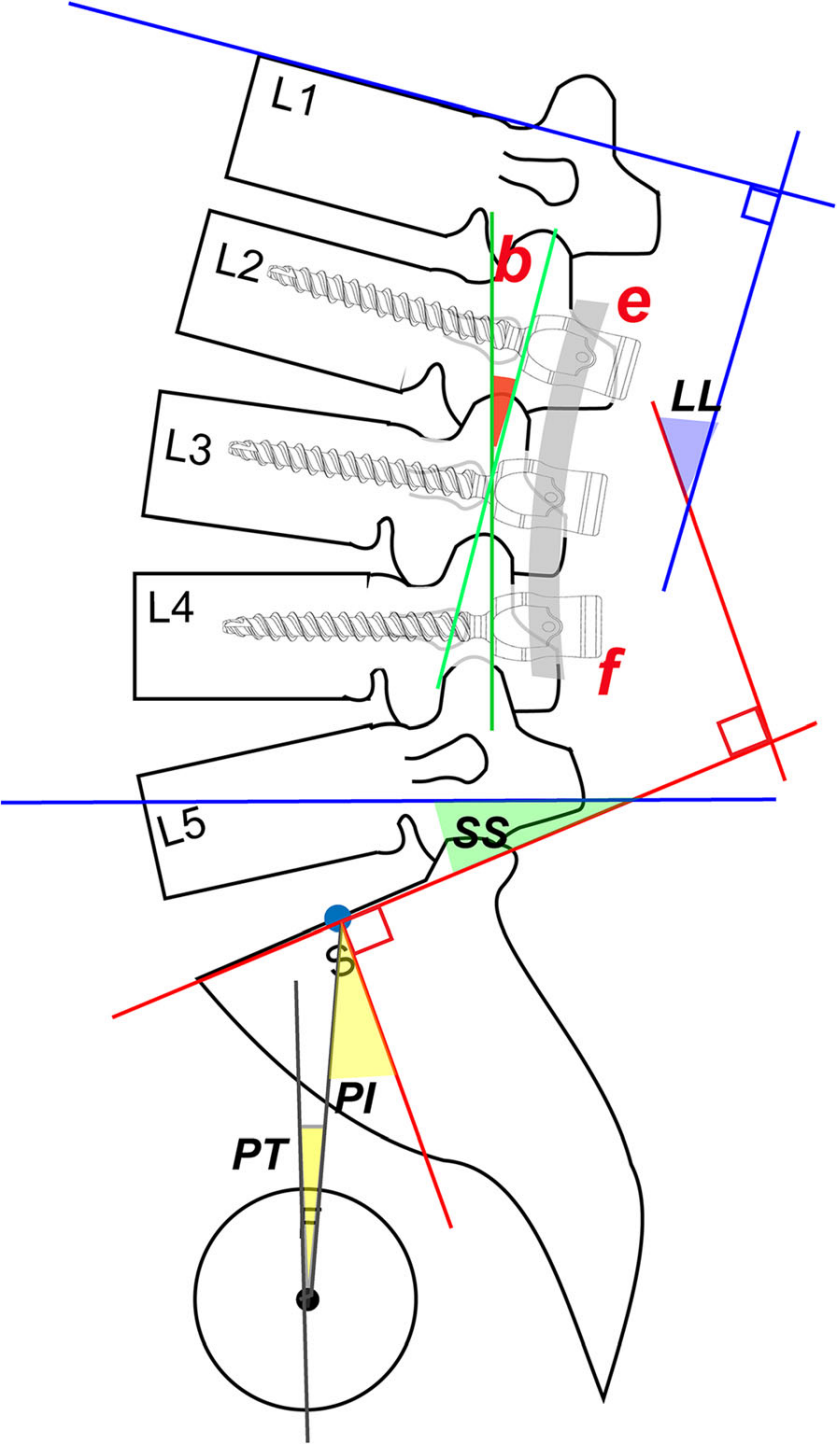
(LL) The angle between the perpendicular lines of the T12 and S1 upper endplates. (TL) The angle between the perpendicular lines of the T10 upper endplate and L2 lower endplates. (PI) The angle between the perpendicular lines of the S1 upper endplate and the line between the midline point of S1 upper endplate and midline point of bilateral caput femoris. (PT) The angle between the plumb line and the line between the midline point of S1 upper endplate and the midline point of bilateral caput femoris. (SS) The angle between the perpendicular line of S1 upper endplate and the horizontal line. The green lines indicate the tangents of the connecting points (point e and point f) of the rod and the upper and lower pedicle screws. Angle b is the angle between the two green lines which is indicated as the bending rod angle in this study

The radiological parameters were measured by two observers, including lumbar lordosis (LL), thoracic kyphosis (TK), pelvic tilt (PT), pelvic incidence (PI), sagittal vertical axis (SVA), and sacral slope (SS). The above parameters were measured referring the previous studies [7, 11, 12] and the measured methods were shown in Fig. 1. The rod bending method was also shown in Fig. 1. ASD was evaluated by examining the height of the intervertebral disk, endplate sclerosis, osteophytes, and spondylolisthesis. The definition of ASD was referred by the previous study [13–15].

**Fig. 2:**
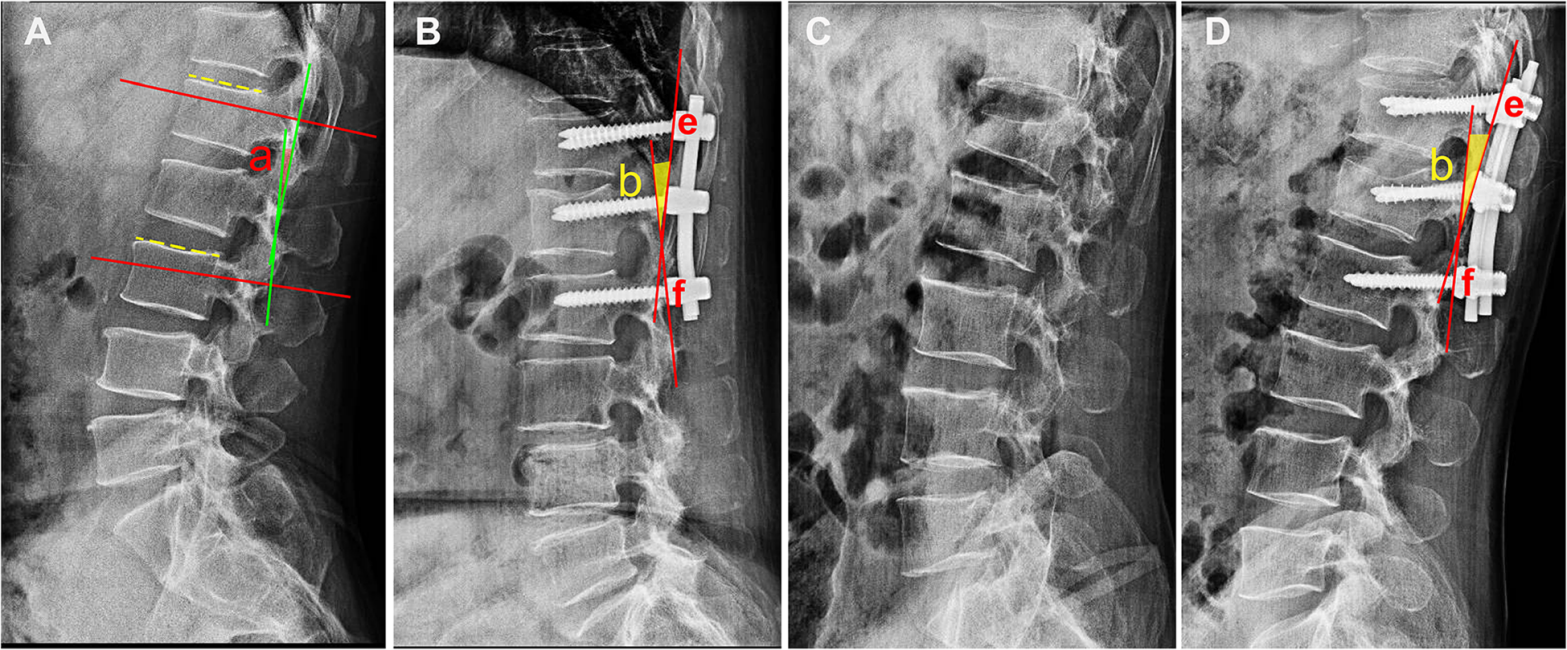
**a** The red lines indicate the parallel lines of the upper and lower endplates. The green lines indicate the vertical lines of the red lines. Angle a is the angle between the two green lines which is indicated as the sagittal Cobb* angle in the study. **b** The red lines indicate the tangents of the connecting points (point e and point f) of the rod and the upper and lower pedicle screws. Angle b is the angle between the two red lines which is indicated as the bending rod angle in this study. **c** The preoperative unstable thoracolumbar fracture lateral radiography. **d** The postoperative unstable thoracolumbar fracture lateral radiography. The red lines indicate the tangents of the connecting points (point e and point f) of the rod and the upper and lower pedicle screws. Angle b is the angle between the two red lines which is indicated as the bending rod angle in this study.

The method of defining the spinal sagittal Cobb* angle was shown in Fig. 2a. The red lines were indicated as the parallel lines of the upper and lower vertebral body end plate. The green lines are perpendicular to the red lines. The spinal sagittal Cobb* angle (angle a) is the angle between the two green lines. The method of bending the connecting rod was shown as Fig. 2b. The bending mark points (point e and point f) of the connecting rod need to be accurately embedded in the U-shaped groove of the upper screw and the lower screw. The angle of the connecting rod was defined as the angle (angle b) between the tangents of point e and point f (the red lines). The preoperative and postoperative unstable thoracolumbar fracture lateral radiographies were shown in Figs. 2c and d.

The frontal and lateral X-ray films in 150 cases of normal thoracolumbar spine were included to measure the normal spinal sagittal Cobb* angle in each segment. The exclusion criteria were as follows: (1) significantly degenerative lordosis, kyphosis, and scoliosis; (2) the “double line shadow” of pedicle or the posterior margin of the vertebral body more than 5 mm; (3) intervertebral space stenosis; and (4) other factors that cause obvious spinal instability.

### Statistical analysis

Statistical analyses were performed using the SPSS.22 statistical software. All values were expressed as means ± standard deviation. *p* value was calculated according to the independent samples *t* test. *p* < 0.05 indicates a statistically significant difference.

## Results

There were no significant differences in the age and gender between the normal and surgery groups (*p* > 0.05). Compared with the age, gender, injury time, and the fracture vertebral, there were no significant differences between the group A, group B, and group C, respectively (*p* > 0.05). The fracture segments in each group were shown in Table 1. The average values of normal sagittal Cobb* angle in each segment were − 5.196 ± 3.318° (T12), 2.279 ± 3.324° (L1), 7.222 ± 2.798° (L2), and 12.417 ± 11.962° (L3), respectively.

The spine-pelvic parameters in normal people, group A, group B, and group C 2 years after surgery were shown in Table 2. The LL in the three groups was 35.20 ± 9.12°, 46.26 ± 9.68°, and 54.24 ± 15.31°, respectively. Compared with the normal group, there were significant differences in group A and group C, respectively (*p* < 0.05). The results were similar in the parameters of TL, PT, and SS. The incidences of SVA > 50 mm in group A, group B, and group C were 23.33%, 12.50%, and 19.23%, respectively. The incidences of SVA > 50 mm in group A and group C were remarkably greater than that in group B (*p* < 0.05), and there was no significant difference in group A and group C (*p* > 0.05). The parameter of PI in three groups was 41.36 ± 12.69°, 44.53 ± 15.27°, and 43.38 ± 9.85°. Compared with the normal group, there was no significant difference in group A, group B, and group C (*p* > 0.05). The incidences of ASD in group A, group B, and group C 2 years after surgery were 21.67%, 13.75%, and 17.95%, respectively. The incidences of ASD in group A and group C were remarkably greater than that in group B (*p* < 0.05), and there was no significant difference in group A and group C (*p* > 0.05).

**Table 2:** The spine-pelvic parameters 2 years after operation

	Group A	Group B	Group C
Spinal parameters			
LL (°)	35.20 ± 9.12*	46.26 ± 9.68	54.24 ± 15.31*
TL (°)	− 29.87 ± 16.38*	− 20.71 ± 13.82	− 11.21 ± 14.45*
SVA > 50 mm, *n*%	23.33	12.50	19.23
Pelvic parameters (°)			
PI (°)	41.36 ± 12.69	44.53 ± 15.27	43.38 ± 9.85
PT (°)	18.49 ± 13.65*	15.71 ± 10.53	23.95 ± 15.51*
SS (°)	18.56 ± 8.47*	26.28 ± 8.55	34.36 ± 9.75*

*The difference was statistically significant

## Discussion

At present, there are few studies on the bending angle of connecting rods in pedicle screw fixation for thoracolumbar fractures. Some studies have confirmed that the bending angle of the connecting rod after thoracolumbar fractures has a significant correlation with the postoperative spinal stability [7, 8, 16–18]. Hongbing and Jia used the normal spinal sagittal Cobb angle as a reference guide for intraoperative bending [10]. The study confirmed the importance of the rod bending angle by measuring the angle relationship between the connecting rod and the screw during the operation. However, this study lacked long-term follow-up after operation. What is more, in their studies, the rod bending angle was the angle between the tangent lines at the two ends of the connecting rod. Another research confirmed that the arc between the connecting points of the connecting rod and the screw is the effective arc [19]. Moufid et al. confirmed that the angle between the screw and the rod, the angle between the screw and the upper endplate, and the distance between the posterior wall and the rod were significantly related to the incidence of adjacent segment degeneration after surgery [7]. This study confirmed that the bending rod angle was correlated with the post-operation spinal stability.

In a small number of studies on the bending angle of the connecting rod, the researchers thought that the angle should almost match the kyphosis angle [20]. A large number of studies have shown that the coronal Cobb angle is an important indicator of the balance of the coronal position of the spine [7, 19–23]. According to previous studies, the sagittal Cobb angle is also an important index used to evaluate the spine sagittal balance [8, 24–26]. In this study, for the single thoracolumbar vertebra fracture, we redefined the sagittal Cobb angle of a single fractured vertebra as the sagittal Cobb* angle, and its measurement method. Our study first measured the sagittal Cobb* angle of each segment of the normal thoracolumbar segment. Then, the post-operation spinal sagittal stability was analyzed in the retrospective research. The results showed that the sagittal Cobb* angle can be used as a reference angle for bending rods. The contact position of the screw and the rod is not the end of the rod, but the contact position of the U-shaped groove of the screw and the rod. Therefore, the curvature of the excess rod on the upper and lower U-shaped grooves cannot maintain the lordosis and kyphosis angle. Therefore, in our study, the sagittal Cobb* angle is the angle between the tangent of the connection point of the upper screw and the rod and the tangent of the connection point of the lower screw and the rod.

In this study, we selected 150 normal adult lateral spine radiographs. By measuring the sagittal Cobb* angle from T12 to L3 vertebral bodies, we obtained the Cobb* angle reference range of each vertebral body. Some studies described the spinal segmental sagittal curvature as “segmental lordosis” [27–29]. The sagittal Cobb* angle in this study describes the lordosis range of three consecutive vertebral bodies. Compared with the LL, the variation in different populations is smaller, and the description of the staged lordosis angle is more accurate. The result showed that when the bending angle of the connecting rod is 4 to 8° greater than the corresponding segment sagittal Cobb angle, the patient’s spinal sagittal stability is the best 2 years after the operation. This result further confirms the feasibility and accuracy of using the sagittal Cobb* angle to guide the bending rod.

In this study, the spine stability parameters and the incidence of ASD 2 years after surgery were used to evaluate the spine sagittal stability. The importance of the spinal sagittal stability after vertebral surgery has been shown in many studies [7, 26, 30, 31]. The spinal sagittal parameters include SVA, LL, and TL. Previous studies have shown that the sagittal stability of the spine decreases when SVA > 50.0 mm. The smaller the value of LL, the higher the incidence of ASD in patients [32]. ASD after lumbar spine surgery is a long-term complication that seriously affects the prognosis of patients. It will cause not only long-term intractable low back pain after surgery, but also some symptomatic ASD that requires secondary surgery [33]. The sagittal imbalance of the spine is one of the main factors leading to ASD [34]. The results showed that when the angle of the bending rod is 4 to 8° greater than the sagittal Cobb* angle, the incidence of spinal imbalance is the lowest, which can maximize the sagittal stability of the spine, and the incidence of ASD is lowest. Although the results showed that the incidence of ASD after operation in group C was not different from that in group B, the results of LL, SVA, PI, and PT in the two groups showed that group B has better spinal sagittal stability. In the comparison of pelvic parameters, the value of PI was not statistically significant in the three groups. Therefore, we believe that the angle of the bending rod has little effect on postoperative PI. The results of PT and SS are consistent with the results of the spine sagittal parameters.

At the beginning of the study, we estimated that using the normal sagittal Cobb* angle to guide the bending rod will achieve the best postoperative results, but the final result shows that the bent rod angle is 4 to 8° greater than the sagittal Cobb* angle to achieve the best effect. To analyze the reason, we consider that the bending angle of the connecting rod is greater than the sagittal Cobb* angle and can resist the loss of the arc of the connecting rod pre-bending caused by the expansion, tightening of the nut, the rotation of the universal screw, early activity, and the increasing age. What is more, the hyperextension of the connecting rod can minimize the incidence of ASD in patients after surgery.

In the thoracolumbar segment, hyperextension fixation is more conducive to the restoration of spine sagittal balance and reduces the incidence of degeneration of the adjacent segment after surgery. Finally, our study proved that the accuracy of the angle of the bent rod is more important for the postoperative spine sagittal balance.

This study has shown obvious advantages in the method of sample grouping, comparison setup, definition and measurement of Cobb* angle, description of effective radian, and method of bending rods. Nevertheless, this study still has some limitations. First, this study included a small sample size during measurement of the normal spinal sagittal Cobb* angle. More sample sizes need to be included in future studies. Second, the study is a retrospective study, with selection bias and loss of follow-up. In future studies, prospective randomized controlled studies can be used to increase the credibility of the results. This study confirmed the influence of the angle of the bent rod on postoperative sagittal spine balance, but there is no further analysis on the influence of factors on the curvature of the bending rod such as the height of the vertebral body after the pre-installation of the connecting rod during the operation and the postoperative activities. Third, the follow-up period of the study was 2 years. Studies have shown that degeneration of the adjacent segment after thoracolumbar fracture surgery mostly occurs 3–5 years after surgery [35, 36]. In future studies, we will reduce the limitations of this study to further confirm the feasibility of the sagittal Cobb* angle to guide the intraoperative bending rod.

## Conclusions

The study confirmed that the sagittal Cobb* angle can be used as a reference angle for bending rods. When the bending angle of the connecting rod is 4 to 8° greater than the corresponding segment sagittal Cobb* angle, the patient’s spinal sagittal stability is the best 2 years after the operation.

## Data Availability

The datasets used and analyzed in this study are available from the corresponding author on reasonable request.

## Abbreviations

LL: Lumbar lordosis
TK: Thoracic kyphosis
PT: Pelvic tilt
PI: Pelvic incidence
SS: Sacral slope
SVA: Sagittal vertical axis
ASD: Adjacent segment degeneration
